# Pathological mechanisms underlying single large‐scale mitochondrial DNA deletions

**DOI:** 10.1002/ana.25127

**Published:** 2018-01-24

**Authors:** Mariana C. Rocha, Hannah S. Rosa, John P. Grady, Emma L. Blakely, Langping He, Nadine Romain, Ronald G. Haller, Jane Newman, Robert McFarland, Yi Shiau Ng, Grainne S. Gorman, Andrew M. Schaefer, Helen A. Tuppen, Robert W. Taylor, Doug M. Turnbull

**Affiliations:** ^1^ Wellcome Centre for Mitochondrial Research, Institute of Neuroscience Newcastle University Newcastle upon Tyne United Kingdom; ^2^ National Health Service Highly Specialised Mitochondrial Diagnostic Laboratory, Newcastle upon Tyne Hospitals National Health Service Foundation Trust Newcastle upon Tyne United Kingdom; ^3^ Department of Neurology and Neurotherapeutics University of Texas Southwestern Medical Center Dallas TX; ^4^ Institute for Exercise and Environmental Medicine of Texas Health Presbyterian Hospital Dallas TX.; ^5^ Present address: Current address for Dr Grady: Garvan Institute Sydney New South Wales Australia; ^6^ Present address: Current address for Dr Rocha: Cardiovascular Division, King's College London British Heart Foundation Centre of Excellence London United Kingdom

## Abstract

**Objective:**

Single, large‐scale deletions in mitochondrial DNA (mtDNA) are a common cause of mitochondrial disease. This study aimed to investigate the relationship between the genetic defect and molecular phenotype to improve understanding of pathogenic mechanisms associated with single, large‐scale mtDNA deletions in skeletal muscle.

**Methods:**

We investigated 23 muscle biopsies taken from adult patients (6 males/17 females with a mean age of 43 years) with characterized single, large‐scale mtDNA deletions. Mitochondrial respiratory chain deficiency in skeletal muscle biopsies was quantified by immunoreactivity levels for complex I and complex IV proteins. Single muscle fibers with varying degrees of deficiency were selected from 6 patient biopsies for determination of mtDNA deletion level and copy number by quantitative polymerase chain reaction.

**Results:**

We have defined 3 “classes” of single, large‐scale deletion with distinct patterns of mitochondrial deficiency, determined by the size and location of the deletion. Single fiber analyses showed that fibers with greater respiratory chain deficiency harbored higher levels of mtDNA deletion with an increase in total mtDNA copy number. For the first time, we have demonstrated that threshold levels for complex I and complex IV deficiency differ based on deletion class.

**Interpretation:**

Combining genetic and immunofluorescent assays, we conclude that thresholds for complex I and complex IV deficiency are modulated by the deletion of complex‐specific protein‐encoding genes. Furthermore, removal of mt‐tRNA genes impacts specific complexes only at high deletion levels, when complex‐specific protein‐encoding genes remain. These novel findings provide valuable insight into the pathogenic mechanisms associated with these mutations. Ann Neurol 2018;83:115–130

Single, large‐scale deletions of mitochondrial DNA (mtDNA) are a common cause of adult mitochondrial disease, accounting for approximately 16% of all adult mtDNA mutations and with an estimated prevalence of 1.5/100,000.^1^ Phenotypically, single large‐scale mtDNA deletions are associated with several clinical syndromes including Pearson syndrome,^2^ Kearns–Sayre syndrome,^3^ and chronic progressive external ophthalmoplegia (CPEO).^4^ A recent prospective study noted ptosis, ophthalmoplegia, muscle weakness, exercise intolerance, and hearing loss as the most common symptoms present at onset.^5^

Although a specific deletion encompassing 4,977bp is commonly reported,^6^ single, large‐scale mtDNA deletions vary in size (between 1.3 and 10kb), removing a number of genes encoding both mt‐tRNAs and structural mRNAs.^7^, ^8^, ^9^, ^10^, ^11^ They are typically mutational events that occur during the replication/repair of the mitochondrial genome and are invariably heteroplasmic in human tissues ‐ a condition where both mutated and wild‐type mtDNA genomes coexist in the same cell. In postmitotic tissues, such as skeletal muscle, the proportion of deleted to wild‐type mtDNA must exceed a reported threshold (50–90%)^9^, ^10^, ^11^, ^12^, ^13^ for the expression of a biochemical defect, leading to a marked mosaicism between different fibers and well‐described cytochrome *c* oxidase (COX)‐deficient, ragged‐red fibers.^13^

Since they were first described,^14^ several groups have tried to understand the pathogenesis of single, large‐scale mtDNA deletions and pinpoint the factors driving disease progression. However, the different mtDNA deletion sizes and phenotypic heterogeneity linked with these mutations have led to conflicting results. Larger mtDNA deletions have been associated with more severe disease and earlier disease onset.^15^, ^16^, ^17^, ^18^, ^19^ Whereas a highly significant,^20^ significant,^15^, ^16^, ^21^, ^22^ weak,^18^, ^23^ or no^24^ correlation has been reported between the percentage of COX‐deficient fibers and the degree of mtDNA heteroplasmy, most studies found no correlation between the biochemical defects and the nature of the deletion (the number of protein‐encoding genes and the complexes affected by the deletion) or deletion size alone.^4^, ^15^, ^20^, ^22^, ^25^ Interestingly, a higher proportion of COX‐deficient ragged‐red fibers was found in patients with deletions encompassing the 3 mtDNA‐encoded COX genes,^26^, ^27^ whereas an isolated complex I deficiency was reported in patients with smaller mtDNA deletions removing only complex I genes.^26^

There are limitations to these studies, which account for the variability in their findings. Respiratory‐deficient muscle fibers were selected based on the sequential COX/succinate dehydrogenase histochemistry, which only assesses complex IV function. Therefore, fibers exhibiting high levels of deficiency involving other respiratory chain complexes might be missed. Despite few exceptions,^16^, ^17^, ^19^, ^26^ patients were also often evaluated as one group, without discriminating deletions of different sizes and locations. Such an analysis cannot determine whether the nature of the deletion influences biochemical and clinical profiles.

In this study, we combined quantitative immunofluorescent^28^ and molecular genetic techniques to investigate the interplay between the nature of the pathological mtDNA deletion and its consequential mitochondrial respiratory chain profile, the threshold for respiratory chain deficiency, and the metabolic profile based on skeletal muscle fiber type.

## Materials and Methods

### Cohort Clinical Characteristics

Vastus lateralis needle biopsies were obtained from 23 patients referred to the National Health Service (NHS) Highly Specialised Mitochondrial Service in Newcastle with a clinical and molecular diagnosis of mitochondrial disease due to a single, large‐scale mtDNA deletion in muscle. All biopsies had previously been characterized to determine the precise size and level of the deletion through mapping mtDNA deletion breakpoints and assessing levels of mtDNA heteroplasmy (or deletion level) in muscle homogenates (Table 1). Ethical approval was granted by the Newcastle and North Tyneside 1 and National Research Ethics Committees (reference 2002/205 “Role of Mitochondrial Abnormalities in Disease”) and by the University of Texas Southwestern Institutional Review Board (study ID: STU 092010‐077, “Exercise Adaptations in Mitochondrial Myopathy: Therapeutic Implications”), and informed consent was obtained from each participant.

**Table 1:** Clinical Information of Patients Included in This Study

Patient	Age, yr^a^	Gender	Clinical Information	mtDNA Breakpoints	mtDNA Deletion Level
Class I: complexes I and IV equally downregulated					
P1	21	F	CPEO, myopathy, cerebellar ataxia, short stature (KSS)	8482–13460	87%
P2	22	F	CPEO	8543–15672	7%
P3	25	M	CPEO, myopathy	8569–14603	78%
P4	29	F	CPEO	8929–13301	53%
P5	36	F	CPEO, myopathy	8577–12983	78%
P6	43	F	CPEO, myopathy	8482–13460	73%
P7	44	F	CPEO, myopathy	9486–13723	81%
P8	48	F	CPEO, myopathy	9498–13739	39%
Class II: complex I more affected than complex IV					
P9	25	F	CPEO	13039–15661	66%
P10	26	F	CPEO, myopathy	10747–15598	71%
P11	26	F	CPEO, myopathy, deafness, diabetes	10946– 15587	83%
P12	39	M	CPEO, myopathy	11262– 15375	81%
P13	40	F	CPEO, myopathy, bulbar weakness, muscle atrophy, pigmentary retinopathy	12113–14421	90%
P14	47	F	CPEO	12112– 14412	70%
P15	50–57	M	CPEO, diabetes	12112– 14412	59%
P16	59	F	CPEO	12211–15556	38%
Class III: complex IV slightly more affected than complex I					
P17	28	F	CPEO, myopathy	6341–13989	33%
P18	31–34	M	CPEO, myopathy	5772–12916	36%
P19	39	F	CPEO	7130–14628	28%
P20	41	F	CPEO, myopathy	6742–13223	19%
P21	59	M	CPEO, myopathy	6002–11221	15%
P22	63	M	CPEO	7128–13992	35%
P23	74	F	CPEO	7205–12090	34%

a Age when biopsied.

CPEO = chronic progressive external ophthalmoplegia; F = female; KSS = Kearns–Sayre syndrome; M = male.

### Oxidative Phosphorylation Quadruple Immunofluorescence

Oxidative phosphorylation (OXPHOS) quadruple immunofluorescence was carried out on transversely orientated frozen muscle sections (10µm) according to an established and validated protocol.^28^ Briefly, the sections were incubated with a cocktail of primary antibodies (COX‐I, porin, NDUFB8, and laminin) followed by incubation with the secondary antibodies (Alexa Fluor 488, 546, biotinylated IgG1 and 750) and subsequently with streptavidin 647. A no‐primary antibody control (only labeled with laminin) was processed for each muscle sample.

The *z* scores for porin (porin_z), NDUFB8 (NDUFB8_z), and COX‐I (COX‐I_z) from individual fibers were derived^28^ and used to infer porin levels (porin_z < −3 standard deviations [SD], very low; −3 SD < porin_z < −2SD, low; −2 SD < porin_z < +2 SD, normal; +2 SD < porin_z < +3 SD, high; porin_z > +3 SD, very high) and both NDUFB8 and COX‐I levels (*z* > −3 SD, normal; −3 SD > *z* > −4.5 SD, intermediate positive; −4.5 SD > *z* > −6 SD, intermediate negative; *z* < −6 SD, negative).

### Fiber Typing Immunofluorescence

Fiber typing immunofluorescence was carried out on transverse muscle sections of 20μm thickness, as previously described.^29^ The sections were incubated with the primary antibody cocktail (mouse IgG2b BA‐F8 [to identify type I fibers, in a 1:100 dilution], mouse IgG1 SC‐71 [type IIa, 1:100], mouse IgM 6H1 [type IIx, 1:15; Developmental Studies Hybridoma Bank, Iowa City, IA], and rabbit IgG laminin [1:50] in 5% normal goat serum [NGS]) followed by incubation with the secondary antibody cocktail (anti‐IgG2b Alexa Fluor 488, anti‐IgG1 Alexa Fluor 546, anti‐IgM Alexa Fluor 647, antirabbit Alexa Fluor750, all diluted 1:200 in 5% NGS). The sections were stored at 4°C for mtDNA preservation, before imaging. To assess the fiber type profile, muscle fibers were visually classified into either type I or type II fibers, based on the presence of myosin heavy chain isoforms.

### Image Acquisition

Fluorescent images were acquired at ×20 magnification using a Carl Zeiss (Oberkochen, Germany) Axio Imager M1 and Zen 2011 (blue edition) software equipped with a motorized stage, a monochrome digital camera (AxioCam MRm), and 488nm (COX‐I or type I), 546nm (porin or type IIa), 647nm (NDUFB8 or type IIx), and 750nm (laminin) wavelength filter cubes. Images were recorded as zvi files and processed by Zen 2011 (blue edition) software using the stitching function.

### Selection and Isolation of Single Skeletal Muscle Fibers for Molecular Genetic Analysis

**Figure 1:**
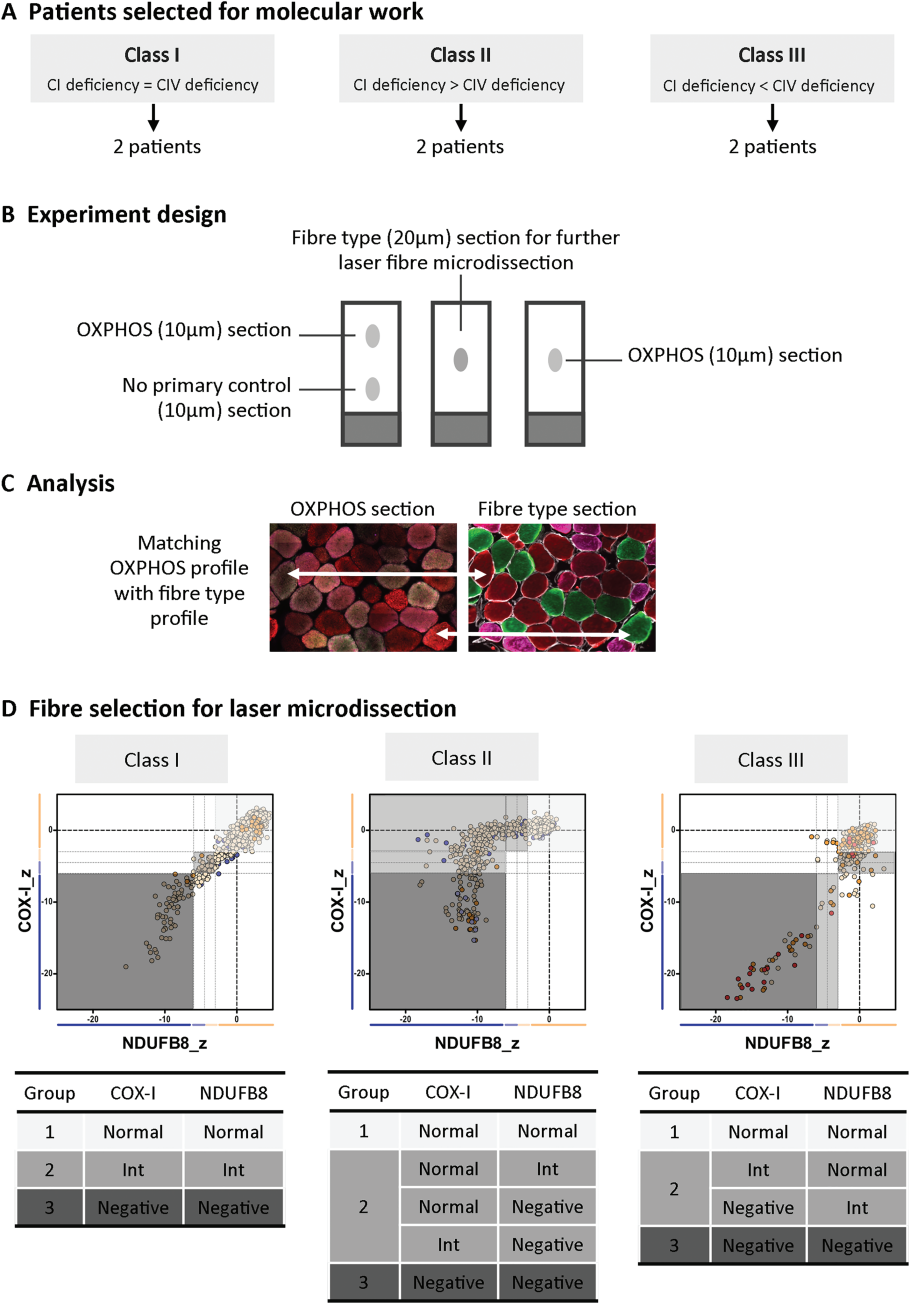
Design of the molecular study. (A) Patients were first classified into different classes (I–III) according to their mitochondrial respiratory chain profile, and then 2 patients from each class were selected for single fiber analysis based on their biopsy size. (B) Four serial muscle sections were taken from each patient's biopsies for oxidative phosphorylation (OXPHOS) and fiber type analysis. (C) The OXPHOS section was sequentially incubated with all primary and secondary antibodies (green = COX‐I, red = porin, purple= NDUFB8), whereas the no primary control section was inbubated only with laminin antibody and all secondary antibodies. The fiber type section was incubated with antibodies detecting type I (green) and type II (red = IIa, purple = IIx) myosin heavy chain. The OXPHOS and fiber type serial sections were manually overlaid to match fibers across sections. (D) Fibers were then selected for microdissection, with careful consideration of the OXPHOS group (1–3) and fiber type. CI = complex I; CIV = complex IV; Int = intermediate.

An outline of the single fiber approach is shown in Figure 1. Following assessment of the OXPHOS deficiency, we noted that patients with single large‐scale mtDNA deletions exhibited distinct mitochondrial respiratory chain profiles. There was either an equal and simultaneous loss of COX‐I (complex IV) and NDUFB8 (complex I), a more pronounced loss of complex I over complex IV, or the opposite, a slightly more pronounced involvement of complex IV over complex I. Therefore, patients were grouped on the basis of that profile, into classes I to III, respectively. Next, 2 patients were selected from each class for further molecular investigations by single fiber analysis based on quality of biopsies and number of fibers available to analyze. Four serial sections were collected from patient skeletal muscle biopsies for OXPHOS and fiber type immunofluorescence protocols. Subsequent to immunofluorescent staining, OXPHOS and fiber type serial sections were aligned and fibers were manually matched across sections to provide a mitochondrial respiratory chain and fiber type profile for each individual myofiber. Selection of muscle fibers for laser microdissection and molecular genetic analysis was carried out as shown in Figure 1D. Muscle fibers were categorized according to the level of complex I and complex IV deficiency into groups (referred to as OXPHOS groups): double‐positive, intermediate, or double‐negative for both complexes I and IV. Subsequently, equal numbers (minimum of 10 fibers whenever possible) of type I and type II fibers were randomly selected from each group. Fiber typing sections were dehydrated through an ethanol gradient, and selected muscle fibers were microdissected using the PALM Microbeam system (Zeiss), as described.^30^

### Real‐Time PCR

In 20 patients (of 23), the level of mtDNA deletion (ie, mutation load) was determined using an established and previously validated duplex *MT‐ND1*/*MT‐ND4* TaqMan real‐time polymerase chain reaction (PCR) assay,^31^ as their mtDNA deletions preserve the *MT‐ND1* site but encompass *MT‐ND4*. A standard curve consisting of a 10‐fold serial dilution of plasmid containing *MT‐ND1* and *MT‐ND4* 30 was loaded in triplicate on each plate. Single fiber lysate stocks were diluted 1:5 in nuclease‐free water and loaded in triplicate onto 3 replicate plates. Absolute copy numbers of *MT‐ND4* and *MT‐ND1* were determined by comparing the average sample Cq against the standard curve. Deletion level was calculated as [1 − (*MT‐ND4*: *MT‐ND1*) * 100]. Total mtDNA copy number was represented by the *MT‐ND1* copy number and wild‐type mtDNA copy number by *MT‐ND4* copy number. New primers and probes were designed to evaluate deletion levels of patients where *MT‐ND4* primer binding sites were preserved. Primers and probes were designed for *MT‐CYB* (forward nt14926–14945; reverse nt14987–14966; probe nt14947–14964) for P9 and P16, and for *MT‐COI* (forward nt6186–6207; reverse nt6249–6229; probe nt6208–6224) for P21 using Primer Express software (Thermo Fisher Scientific, Waltham, MA). These substituted *MT‐ND4* primers and probes in the aforementioned protocol.

### Statistical Analysis

OXPHOS statistical analyses were carried out using a newly developed website (http://research.ncl.ac.uk/mitoresearch/) and R version 3.3.2.^32^ For comparison of deletion levels, copy number, and threshold between fiber groups, the Mann–Whitney *U* and paired *t* tests were applied to assess the difference between respiratory‐normal fibers (OXPHOS group 1) and either the respiratory‐intermediate (OXPHOS group 2) or respiratory‐negative fibers (OXPHOS groups 3).

## Results

### Genes Affected by the mtDNA Deletion Modulate the Mitochondrial Respiratory Chain Profile

**Figure 2:**
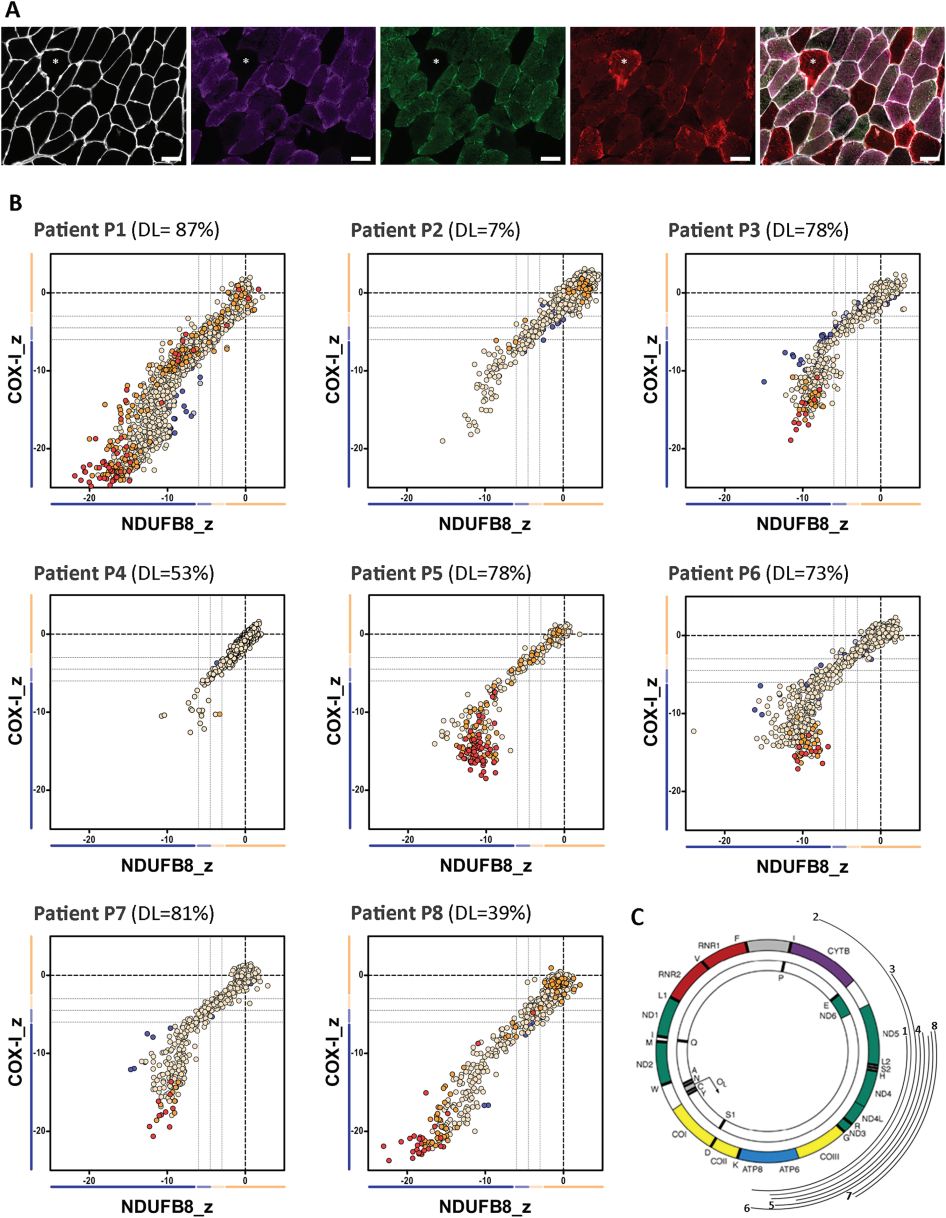
The mitochondrial respiratory chain profile and genotype from patients with single, large‐scale mtDNA deletions grouped in class I, showing both complexes I and IV equally affected. (A) The quadruple immunofluorescence (white = laminin [750nm], purple = NDUFB8 [647nm], green = COX‐I [488nm], red = porin [546nm]) was performed on 10µm sections and highlights *(asterisks)* the presence of fibers with both complex I and IV affected; scale bars measure 50µm. (B) Graphs show complex I and IV expression profile from patients: P1 (n = 1,448 fibers analyzed), P2 (n = 1,261), P3 (n = 631), P4 (n = 1,228), P5 (n = 388), P6 (n = 853), P7 (n = 609), and P8 (n = 1,309). DL indicates the mtDNA deletion load (proportion of deleted over wild‐type mtDNA) determined in muscle homogenates. Each dot represents an individual muscle fiber color coded according to its mitochondrial mass (blue = very low, light blue = low, beige = normal, orange = high, red = very high). Thin black dashed lines indicate the standard deviation limits for the classification of fibers; lines next to x‐ and y‐axes indicate the levels of NDUFB8 and COX‐I, respectively (beige = normal, light beige = intermediate(+), light blue = intermediate(−), blue = negative). Bold dashed lines indicate the mean expression level of normal fibers. (C) Location and size of the mtDNA deletion from individual patients (inside arc = P1 to outside arc = P8).

**Figure 3:**
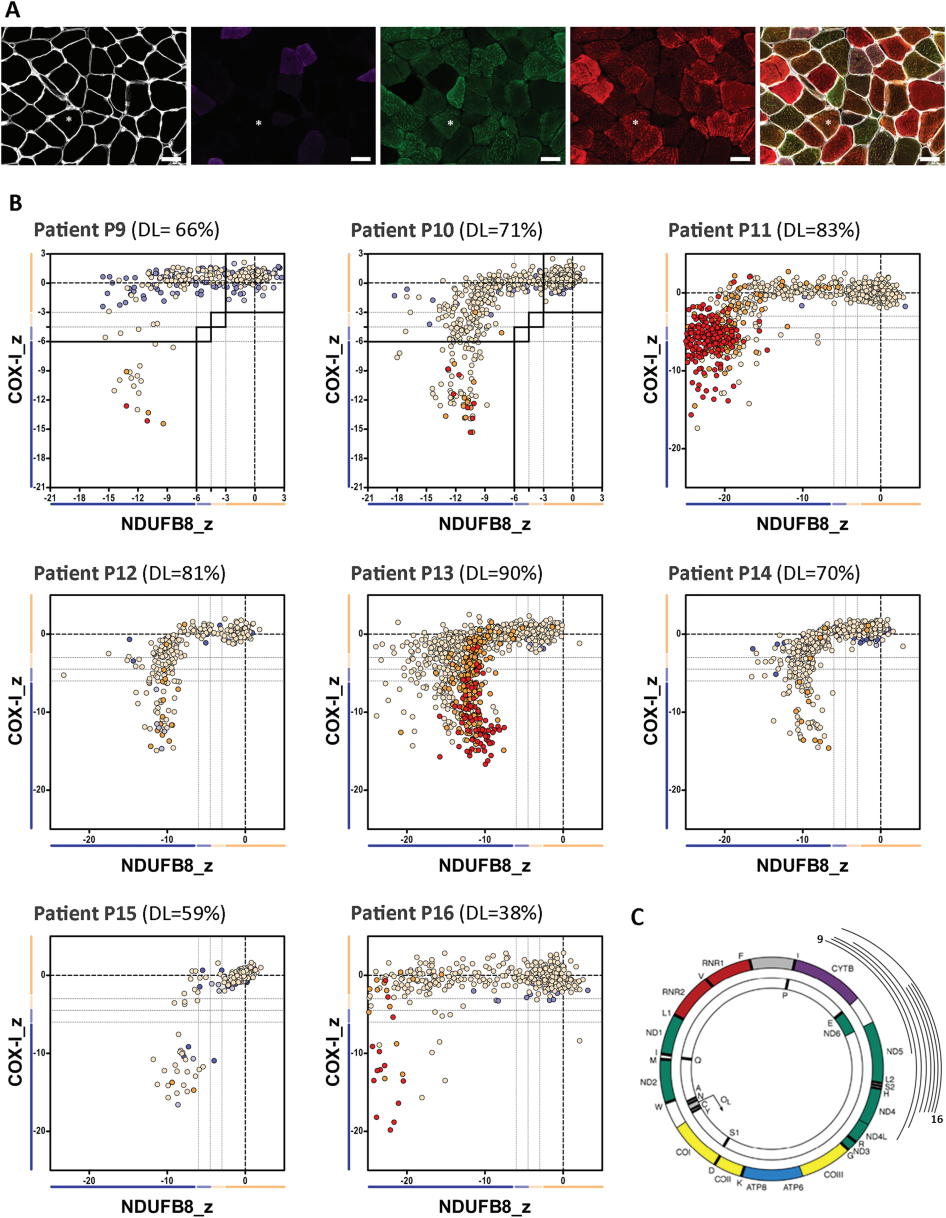
The mitochondrial respiratory chain profile and genotype from patients with single, large‐scale mtDNA deletions grouped in class II, showing a more pronounced involvement of complex I over complex IV. (A) The quadruple immunofluorescence (white = laminin, purple = NDUFB8, green = COX‐I, red = porin) was performed on 10µm sections, and highlights *(asterisks)* the presence of fibers with a more pronounced loss of complex I; scale bars measure 50µm. (B) Graphs show complex I and IV expression profile from patients: P9 (n = 322 fibers analyzed), P10 (n = 579), P11 (n = 1,804), P12 (n = 272), P13 (n = 737), P14 (n = 606), P15 (n = 546), and P16 (n = 283). DL indicates the mtDNA deletion load (proportion of deleted over wild‐type mtDNA) determined in muscle homogenates. Each dot represents an individual muscle fiber color coded according to its mitochondrial mass (blue = very low, light blue = low, beige = normal, orange = high, red = very high). (C) Location and size of the mtDNA deletion from individual patients (inside arc = P9 to outside arc = P16).

**Figure 4:**
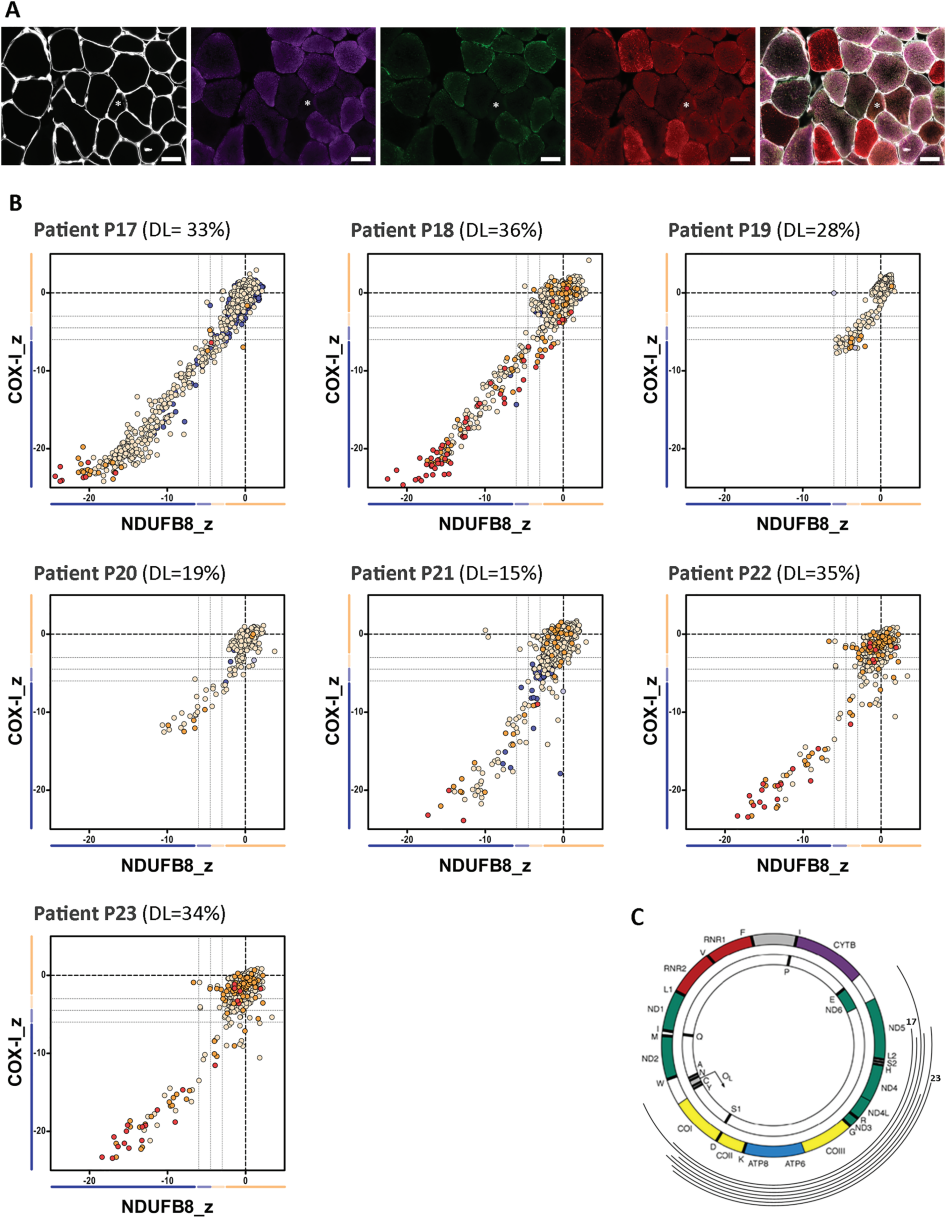
The mitochondrial respiratory chain profile and genotype from patients with single, large‐scale mtDNA deletions grouped in class III, showing a more pronounced involvement of complex VI over complex I. (A) The quadruple immunofluorescence (white = laminin, purple = NDUFB8, green = COX‐I, red = porin) was performed on 10µm sections, and highlights *(asterisks)* the presence of fibers with a more pronounced loss of complex IV; scale bars measure 50µm. (B) Graphs show complex I and IV expression profile from patients: P17 (n = 1,400 fibers analyzed), P18 (n = 764), P19 (n = 841), P20 (n = 283), P21 (n = 1,333), P22 (n = 730), and P23 (n = 1579). DL indicates the mtDNA deletion load (proportion of deleted over wild‐type mtDNA) determined in muscle homogenates. Each dot represents an individual muscle fiber color coded according to its mitochondrial mass (blue = very low, light blue = low, beige = normal, orange = high, red = very high). (C) Location and size of the mtDNA deletion from individual patients (inside arc = P17 to outside arc = P23).

Previous results from our group have shown that patients harboring single, large‐scale mtDNA deletions show decreased levels of both complex I and complex IV, in line with a disorder of generalized mitochondrial translation.^28^ However, as there seemed to be some differences between patients in the degree of complex I and complex IV declines, we decided to investigate a larger group of patients. We performed quadruple immunofluorescence on skeletal muscle biopsies from 23 genetically characterized patients (Figs 2A, 3A, and 4A), and identified 3 distinct mitochondrial respiratory chain profiles (see Fig 2B, 3B, and 4B); consequently, patients were further categorized into 1 of 3 classes: class I (P1–P8), with equal and simultaneous downregulation of both complex I and complex IV; class II (P9–P16), with an early and more pronounced loss of complex I over complex IV; and class III (P17–P23), with slightly more severe complex IV deficiency than complex I deficiency. mtDNA deletion level varied across the whole group of patients (see Table 1 and Figs 2B, 3B, and 4B), regardless of the class, suggesting that, unlike the magnitude of deficiency, the mitochondrial respiratory chain profile is not influenced by the proportion of mutated mtDNA species.

To explore the underlying pathological processes determining the mitochondrial respiratory chain profiles, we then examined the nature of the mtDNA deletions more closely (ie, the size of the deletion, the number of genes affected (both protein‐coding and tRNAs), and the respiratory chain complexes affected by the deletion). Interestingly, the mtDNA deletion sizes segregated by the deletion class; the smallest deletions were identified in patients from class II and the largest deletions in patients from class III (data not shown). We then analyzed each class of patient based on the precise molecular defect as determined by mtDNA breakpoint mapping (see Table 1 and Figs 2C, 3C, and 4C). In all patients from class I, individual mtDNA deletions consistently removed 5 to 6 tRNA genes, 4 to 5 complex I genes, and 1 complex IV gene; complex III and V genes were occasionally removed. By contrast, in class II, deletions only removed 3 to 4 tRNA genes, 3 to 4 complex I genes, and occasionally 1 complex III gene; all complex IV genes were preserved, offering a compelling explanation for the earlier onset of complex I deficiency seen in the respiratory chain profiles of all 8 patients. In class III, deletions removed 6 to 8 tRNA genes, 2 to 4 complex I genes, 2 complex V genes, and all complex IV genes. Removal of all mtDNA genes encoding complex IV subunits by these deletions could explain the slightly greater deficiency of complex IV over complex I in all 7 patients. Interestingly, Patients P4, P5, P6, and P7 (class I) and both P21 and P23 (class III) all have 5 tRNA genes removed, suggesting that the number of tRNA genes removed by the deletion is not a determinant of mitochondrial respiratory chain profile classification.

### Increasing mtDNA Deletion Level Is Associated with the Severity of Complex I and Complex IV Deficiency

An association between genotype and mitochondrial respiratory chain phenotype having been established, 2 patients from each class (class I: P2, P8; class II: P13, P15; and class III: P17, P18) were selected for further single cell investigations based on the size of the biopsy available (see Fig 1). The OXPHOS immunofluorescence assay was repeated together with fiber typing in serial sections, to account for any variability between oxidative (type I) and glycolytic (type II) fibers. Single muscle fibers (n = 75–164) with varying degrees of complex I and IV deficiency were then selected for laser microdissection and the proportion of mutated mtDNA in each myofiber was determined by real‐time PCR.

In all 6 patients, mtDNA deletion level was increased in fibers with higher levels of OXPHOS deficiency (Table 2). Fibers positive for both complex I and complex IV immunoreactivity (OXPHOS group 1), deemed respiratory‐”normal,” harbored variable deletion loads, as previously reported.^9^ In contrast, fibers classed as negative for both complex I and complex IV consistently showed the highest mtDNA deletion levels, with a notably narrow range of deletion level (see Table 2). The difference in deletion level between double‐positive and double‐negative fibers was statistically significant both within the individual patients when comparing the groups of fibers (*p* < 0.0001, Mann–Whitney *U* test), and as a patient cohort when comparing the median deletion levels of each fiber group (*p* < 0.0001, paired *t* test, n = 6).

**Table 2:** mtDNA Deletion Level, Total Copy Number, and Wild‐Type Copy Number in Single Fibers from Patients Selected for Molecular Investigations

	Class I		Class II		Class III	
	P2	P8	P13	P15	P17	P18
Deletion level, median % (CV)						
Normal	19.7 (88.7)	6.1 (159.2)	56.0 (48.5)	14.7 (84.8)	4.4 (143.0)	13.1 (98.8)
Intermediate^a^	78.2 (6.5)^b^	28.6 (86.6)^c^	91.2 (14.2)^b^	83.5 (19.5)^b^	78.2 (31.2)^b^	85.4 (39.1)^c^
Negative	89.2 (12.5)^b^	92.2 (21.5)^b^	95.2 (2.2)^b^	94.8 (17.0)^b^	96.4 (21.3)^b^	94.4 (5.3)^b^
Total copy number*, median (SD)						
Normal	9.3 (45.9)	3.5 (3.2)	8.0 (8.3)	4.6 (3.3)	6.2 (3.2)	13.3 (25.2)
Intermediate^a^	10.5 (6.1)	4.3 (8.2)	14.2 (10.7)^c^	5.0 (7.9)^c^	12.0 (5.9)^b^	20.1 (27.7)
Negative	13.3 (20.9)	10.3 (15.9)^b^	16.0 (22.5)^b^	8.8 (9.0)	30.3 (22.1)^b^	38.5 (52.6)^b^
Wild type copy number*, median (SD)						
Normal	6.3 (46.5)	3.1 (2.3)	3.9 (6.3)	2.9 (3.5)	5.7 (2.2)	11.4 (9.0)
Intermediate^a^	2.1 (1.8)^c^	1.9 (2.0)^c^	1.7 (2.2)^b^	1.1 (0.9)^b^	2.8 (1.8)^b^	3.1 (3.1)^b^
Negative	1.3 (1.7)^b^	0.7 (1.0)^b^	0.9 (0.3)^b^	0.4 (0.5)^b^	1.1 (1.9)^b^	2.4 (2.5)^b^

Mann–Whitney *U* test was used to assess the difference in mtDNA deletion level and copy number between OXPHOS groups. Fibers assessed (n = normal/intermediate/negative): P2 (n = 44/5/26), P8 (n = 41/29/42), P13 (n = 25/95/44), P15 (n = 29/38/24), P17 (n = 34/48/40), P18 (n = 63/21/25).

*Total and wild type mtDNA copy number are presented as copies per square micrometer.

a Fibers with intermediate levels of oxidative phosphorylation deficiency were grouped together in patients belonging to classes II and III.

b *p* ≤ 0.0001.

c *p* ≤ 0.05.

CV = coefficient of variation; SD = standard deviation.

The remaining intermediate groups harbored median deletion levels between those observed for normal and negative groups (see Table 2, “intermediate”). The range of deletion levels in these fibers is large, as they encompass those with slight biochemical deficiency (eg, complex I intermediate and complex IV normal) to more severe deficiency (eg, complex I negative and complex IV intermediate). These fibers represent those cells in transition from “healthy” to fully respiratory‐negative and, therefore, offer valuable insight into the changing genetic and biochemical status of fibers over this phase. Patient 8 demonstrated a much larger number of intermediate fibers with low levels of mtDNA deletion for reasons that are not readily apparent.

### Threshold Level for Complex I and Complex IV Deficiency Is Regulated by the Position of the mtDNA Deletion

**Figure 5:**
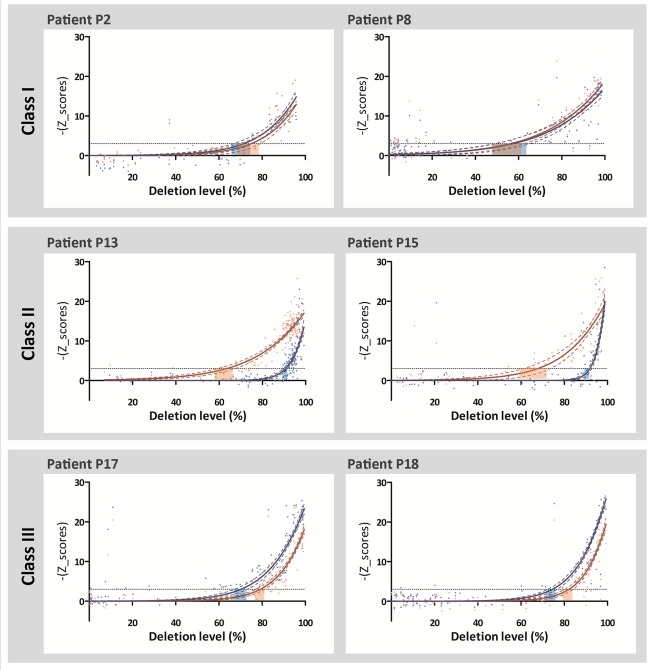
The thresholds for complex I and complex IV deficiency are modulated by the genes covered by the mtDNA deletion. Graphs plot the inverted *z* score for NDUFB8 *(red dots)* and inverted *z* score for COX‐I *(blue dots)* against the deletion level from individual muscle fibers. In class I, the thresholds for complex I (CI) and complex IV (CIV) are not significantly different in both patients (P2: CI threshold = 74.5%, CIV threshold = 71.0%; P8: CI threshold = 55.5%, CIV threshold = 56.8%). However, in class II, complex I has a lower threshold for deficiency (P13: CI threshold = 62.7%, CIV threshold = 90.6%; P15: CI threshold = 67.2%, CIV threshold = 91.7%), whereas in class 3, complex IV has a lower deficiency threshold (P17: CI threshold = 81.9%, CIV threshold = 74.1%; P18: CI threshold = 78.9%, CIV threshold = 69.8%). Red and blue dashed lines indicate the 95% confidence interval of the fitted curves. Black dashed lines (−y = 3) indicate the threshold for deficiency.

To further investigate the relationship between the deletion level and the extent of complex I and complex IV deficiency, we plotted the inverted *z* scores of COX‐I (blue dots) and NDUFB8 (red dots) from each muscle fiber against its deletion level (Fig 5); data transformation was essential to fit a logarithmic regression and model the data. Next, we determined the threshold level for both complexes by deriving the deletion level at *z* score (COX‐I/NDUFB8) = −3 SD using the regression model. As illustrated in Figure 5, the individual thresholds for both complex I and complex IV deficiency were shown to vary according to the class of deletion. For class I deletions, the threshold levels for both complex I and complex IV deficiency were not significantly different in either P2 or P8. However, in both patients tested with class II deletions, complex I displayed a significantly lower threshold for biochemical deficiency than complex IV (P13 and P15; average complex I threshold = 65.0%; average complex IV threshold = 91.2%). Furthermore, we observed the opposite finding in patients with class III deletions, whereby complex IV showed a lower threshold for deficiency (P17 and P18; average complex I threshold = 80.4%; average complex IV threshold = 72.0%).

### Increased Biochemical Deficiency Is Associated with Increased Total and Decreased Wild‐Type mtDNA Copy Number

To determine whether mtDNA copy number played a role in the distinct biochemical deficiency observed between classes of deletions, we determined total mtDNA copy number, presented as copies per square micrometer, using real‐time PCR. As for analysis of the deletion level, fibers were grouped according to the extent of immunoreactive complex I and complex IV deficiency. Total mtDNA copy number increased in line with the severity of OXPHOS deficiency in all patients (see Table 2, *p* < 0.0001, Mann–Whitney *U* test between respiratory‐normal fibers and respiratory‐negative fibers, n = 6). This increase in copy number was threshold‐dependent, with high levels of total mtDNA when both complexes were deficient (data not shown). P2 showed minimal differences in total mtDNA copy number across the different OXPHOS groups, but this patient had the lowest homogenate deletion level (7%) and had some respiratory‐normal fibers with high total mtDNA copy number. The median total mtDNA copy number for each patient was not related to mtDNA deletion class. As opposed to total copy number, wild‐type mtDNA copy number was consistently and significantly shown to decrease with increased levels of OXPHOS deficiency in all patients (see Table 2, *p* < 0.0001, Mann–Whitney *U* test between respiratory‐normal fibers and respiratory‐negative fibers, n = 6). Furthermore, this decrease in wild‐type copy number was also threshold‐dependent, where fibers deficient in both complexes had lower wild‐type mtDNA copy number (data not shown).

### Type II Fibers Accumulate OXPHOS Deficiency to a Greater Extent Than Type I Fibers

To explore whether there was an effect of muscle fiber type on OXPHOS deficiency, we investigated whether there was predominance of one fiber type over the other in these patients. There were no consistent findings; 3 patients showed a larger proportion of type I fibers (P2, P8, and P17), whereas the others showed a predominance of type II fibers (P13, P15, and P18; data not shown).

**Figure 6:**
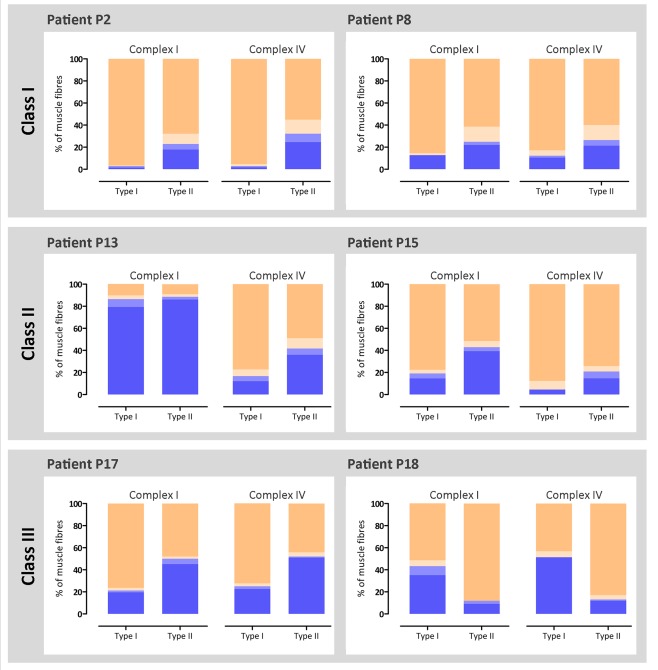
Oxidative phosphorylation (OXPHOS) deficiency in different fiber types. OXPHOS and fiber type immunofluorescence were performed in serial sections, and both profiles were matched for each muscle fiber. Bar graphs show the percentage of fibers with normal (beige), intermediate(+) (light beige), intermediate(−) (light blue), and negative (blue) levels of complex I and complex IV, in type I and type II (including type IIa, type IIx, and/or fibers coexpressing IIa and IIx). Fibers counted (n = type I/type II fibers): P2 (n = 325/118), P8 (n = 494/277), P13 (n = 324/331), P15 (n = 89/163), P17 (n = 464/253), and P18 (n = 37/176).

Next, we combined all the OXPHOS and fiber type data to investigate whether type I or type II muscle fibers showed different susceptibilities to accumulation of respiratory chain deficiency; a classification of muscle fibers in terms of OXPHOS deficiency, stratified by fiber type, is depicted in Figure 6. With the exception of P18, type II fibers were consistently shown to harbor higher proportions of complex I and complex IV deficient fibers than type I fibers in the same patient; the reason for this difference with P18 is unknown. The genetic analysis of individual muscle fibers revealed no consistent difference in mtDNA deletion level or mtDNA copy number (total or wild‐type) between fiber types (data not shown). It is noteworthy that the differences in the level of complex I and complex IV deficiency observed in patients with different classes of mtDNA deletion were observed in both type I and II fibers.

## Discussion

Elucidating pathogenic mechanisms associated with single, large‐scale mtDNA deletions is challenging due to the genetic heterogeneity. Using a recently validated quadruple immunofluorescent assay that allows accurate assessment of complex I and complex IV protein levels in individual muscle fibers,^28^ we demonstrate a clear link between the nature (the number of protein‐encoding genes and the complexes affected by the deletion) of the mtDNA deletion and the biochemical profile observed within individual skeletal muscle fibers. By combining immunofluorescent and quantitative molecular genetics approaches, we show that the thresholds for the expression of both complex I and complex IV deficiency are highly influenced by the number of protein‐coding genes for each complex included within the mtDNA deletion.

### Mitochondrial Respiratory Chain Profiles

All patients with single, large‐scale mtDNA deletions assessed in this study showed a combined complex I and IV deficiency, consistent with the removal of several mt‐encoded structural subunits and mt‐tRNAs, as previously reported.^18^, ^20^, ^22^, ^33^ However, 3 distinct respiratory chain profiles were observed, suggesting differential rates of COX‐I and NDUFB8 decline. On this basis, patients were assigned to 1 of 3 classes: class I with equal and simultaneous decrease of both complex I and IV; class II with more pronounced loss of complex I than complex IV; and class III with slightly higher involvement of complex IV deficiency. In all 3 groups, a variable number of mt‐tRNA genes and complex I genes were removed. However, the number of deleted complex IV genes differed between classes; in class I, only 1 (*MT‐CO3*) complex IV gene was removed, whereas in class II all complex IV genes (*MT‐CO1*, *MT‐CO2*, and *MT‐CO3*) were preserved, potentially explaining the predominant biochemical complex I deficiency associated with this class of mtDNA deletion. Conversely, all complex IV genes were removed in class III, where complex IV deficiency is more severe than complex I deficiency. These results explain many of the findings of previous reports of isolated complex I or isolated complex IV deficiencies measured biochemically^14^, ^15^, ^18^, ^33^, ^34^ in patients with either small deletions compromising only complex I genes (ie, class II deletions) or larger mtDNA deletions that encompass all mtDNA‐encoded complex IV genes (ie, class III deletions), respectively.^26^ We did not have any patients in whom only complex IV genes were deleted.

### mtDNA Deletion Level and OXPHOS Deficiency

COX‐deficient muscle fibers in patients with single, large‐scale mtDNA deletions have been consistently shown to accumulate high levels of mtDNA deletions.^27^, ^35^, ^36^ Accordingly, we have shown that deletion level increases with higher degrees of OXPHOS deficiency across a total of 673 fibers from 6 patients. Whereas the deletion level recorded in deficient fibers was consistently high among all patients (>85%), the mean deletion level recorded in 236 respiratory‐normal fibers was 22.6% (SD = 23.4%), consistent with others studies recording an average deletion level of 21 to 31%.^9^, ^12^, ^13^ The variable deletion levels of the respiratory‐normal fiber groups recorded in the 6 patients reported here (and others in previous studies) reflect their different homogenate mtDNA deletion levels.

### Pathological Threshold for OXPHOS Deficiency

In this study, we show for the first time that the threshold required for the expression of a respiratory chain deficiency is different for complex I and complex IV, and is affected by the site of the deletion. For class I deletions, where complex I and complex IV levels appear to be equally affected, threshold levels for these complexes are similar. However, for class II deletions, the threshold level for complex I (63–67%) is considerably lower than for complex IV (91–92%), observed alongside a predominant complex I deficiency. For class III deletions, complex IV deficiency occurs at a lower level of mtDNA deletion (70–74%) than complex I deficiency (79–82%), suggesting that the absence of 3 subunits of complex IV has a greater effect on the assembly of the complex IV holoenzyme than the loss of a variable number of complex I subunits on complex I assembly. A lack of histochemical assays to specifically assess complex I (NADH:ubiquinone oxidoreductase) activity has hampered the investigation of complex I deficiency at a single muscle fiber level, and therefore it is not surprising that many studies documented high levels of deletion in COX‐normal ragged‐red fibers.^20^, ^27^ To overcome these limitations, we took advantage of a newly developed assay to quantify complex I and complex IV protein deficiency in single fibers.

### Pathogenesis of Single, Large‐Scale mtDNA Deletions

The pathogenic mechanisms associated with single, large‐scale mtDNA deletions have been long debated. The findings of the current study highlight a more prominent role for protein‐encoding genes in the pathogenesis of single, large‐scale mtDNA deletions, as there is a clear link between the deletion of genes encoding subunits of both complexes I and IV and the respiratory chain dysfunction at the cellular and tissue level. For instance, in class II deletions, where only complex I and tRNAs genes are lost, the threshold for complex IV is substantially higher. Interestingly, this threshold of approximately 90% is similar to those recorded in other mtDNA defects caused by pathogenic variants in mt‐tRNA genes,^37^, ^38^, ^39^, ^40^ suggesting that complex IV deficiency in this class of deletions occurs solely due to a translational defect following removal of tRNA gene sequences. These findings are in line with a report by Hammans and colleagues, who also observed a relationship between the location of mtDNA deletion and the biochemical defect, and therefore the determinant role of mtDNA‐encoded COX genes when removed by the deletion.^26^ An alternative theory suggested a translational defect as the primary driver of pathogenesis.^10^, ^41^ However, if that were so, removal of a greater number of mt‐tRNA genes would have an impact on the threshold. Consequently, we would expect to find the lowest pathological threshold in class III deletions (5–10 mt‐tRNA genes deleted) followed by class I deletions (5–6 mt‐tRNA) and the highest pathological thresholds in class II deletions (3–4 mt‐tRNA), which does not occur. Finally, in all patients, high levels of deletion are associated with low levels of wild‐type mtDNA and high levels of total mtDNA, as previously observed,^42^ and consistent with our hypothesis that haploinsufficiency of protein subunits is the cause of the biochemical defect.

### Effect of Muscle Fiber Types

Although *vastus lateralis* normally has type II fiber predominance, there was no clear predominance of either fiber type across patient biopsies. Studies on the metabolic profile of muscle fibers in patients with OXPHOS deficiency are sparse and sometimes contradictory, reporting either no fiber type predominance,^21^ a type I predominance,^15^, ^43^, ^44^, ^45^, ^46^ or a type II predominance.^47^ Interestingly, we found higher respiratory chain deficiency in type II fibers over type I in 5 of 6 patients. The underlying mechanisms behind this observation are not currently fully understood. Our data collected from single fibers do not provide further clarity, as there was little difference in mtDNA deletion level or copy number between fiber types. Nevertheless, it is important to highlight that the single fibers analyzed consisted of only a small fraction of all muscle fibers that were fiber typed (50% in P15 and P18, <25% in remaining patients). The fibers were selected on their OXPHOS immunofluorescence profile, and therefore might not be representative of the biopsy.

### Clinical Phenotypes

Although we have found clear differences in the biochemical profile of the 3 different deletion classes, our numbers at present do not allow us to infer much in terms of clinical phenotypes. We have studied a limited number of patients in detail and, except for 1 patient, all had a CPEO phenotype. Previous studies from our cohort have used statistical modeling to demonstrate that a variety of outcome measures such as age at onset of symptoms and progression of disease burden, as measured by the Newcastle Mitochondrial Disease Adult Scale, are significantly correlated (*p* < 0.05) with the size of the deletion, the mtDNA deletion level in skeletal muscle, and the position of the mtDNA deletion within the genome.^16^ With this new information about the presence of different biochemical profiles associated with different deletions, it will be of interest to determine whether there is any link between the biochemical defect and the clinical phenotype.

### Conclusions

This work represents the largest study performed in single muscle fibers from patients with single, large‐scale mtDNA deletions in which a combination of quantitative molecular genetics and immunofluorescent techniques were applied to improve our understanding of the molecular pathogenesis of the focal respiratory chain deficiency detected in patient muscle. We show a clear relationship between the mtDNA genes removed by the deletion and (1) the mitochondrial respiratory chain profile of muscle fibers in patients with CPEO and (2) the threshold for complex I and complex IV deficiency. This study has yielded important insights into the mechanisms underlying single large‐scale mtDNA deletions, as we demonstrate that the removal of protein‐encoding genes is directly linked to the expression and severity of the consequential respiratory chain deficiency. Furthermore, specific complexes of the respiratory chain are measurably impacted by tRNA gene removal only when their respective protein‐encoding genes are unaffected.

## Author Contributions

M.C.R., R.W.T., and D.M.T. contributed to the conception and design of the study; M.C.R., H.S.R., J.P.G., N.R., R.G.H., J.N., G.S.G., R.M., Y.S.N., A.M.S., E.L.B., H.A.T and L.H. contributed to the acquisition and analysis of data; M.C.R., H.S.R., and D.M.T. contributed to drafting the text and preparing the figures.

## Potential Conflicts of Interest

Nothing to report.

## Supporting information

Supporting Information
