# Supplementary material for: Pathological mechanisms underlying single large‐scale mitochondrial DNA deletions
Source: Ann Neurol. 2018 Jan 24;83(1):115–30. doi: 10.1002/ana.25127 (PMC5893934; doi:10.1002/ana.25127)
Supplement: Supplementary file 1 — Supporting Information [file ANA-83-115-s001.docx]

**
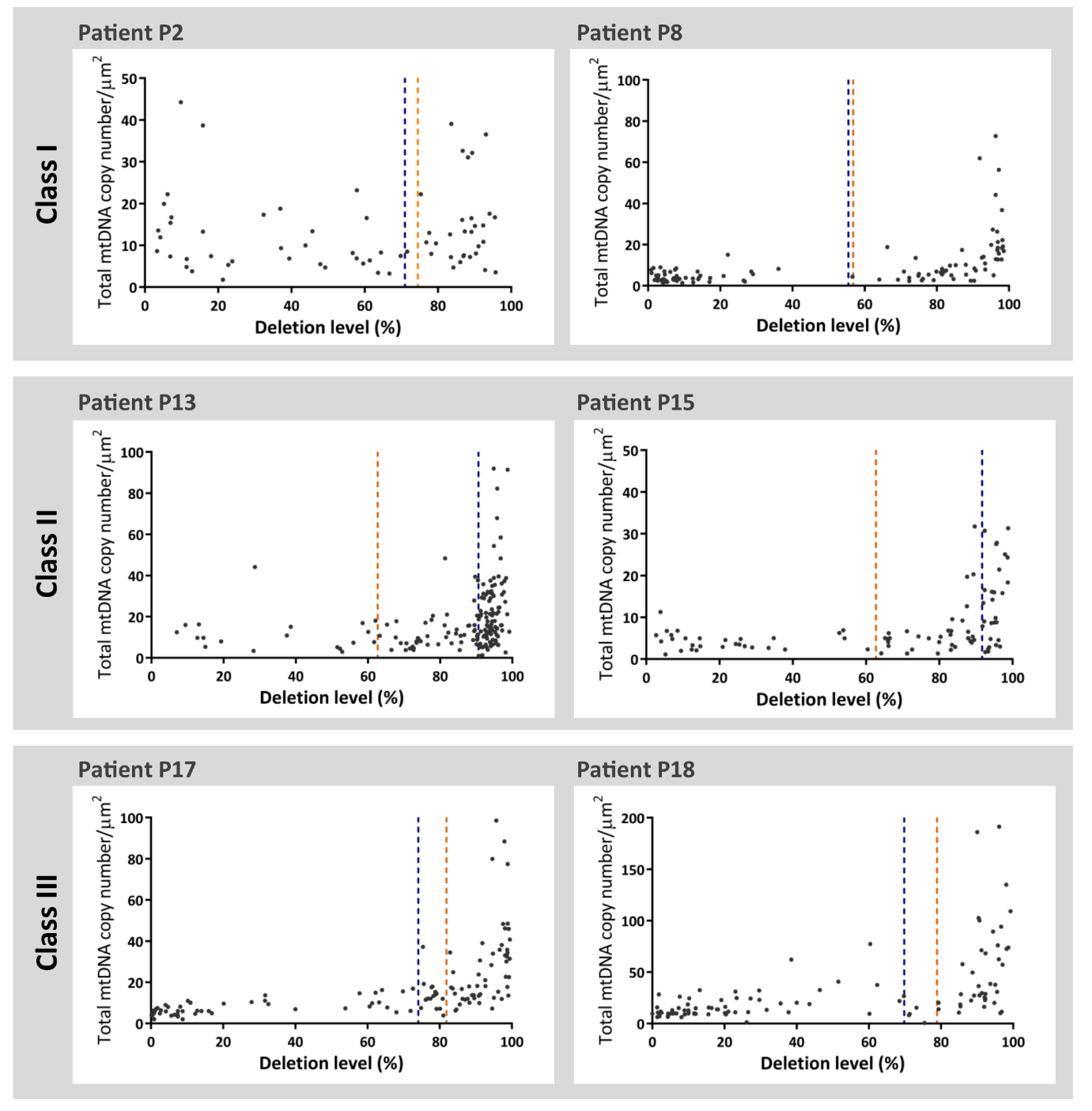
**

**Supplementary material for revision #1: Total mtDNA copy number increases after the biochemical threshold has been reached.** Deletion level and total mtDNA copy number were plotted for each fibre. Each black dot represents one fibre. The previously determined biochemical threshold levels for complex I and IV are shown as orange and blue dashed lines, respectively.


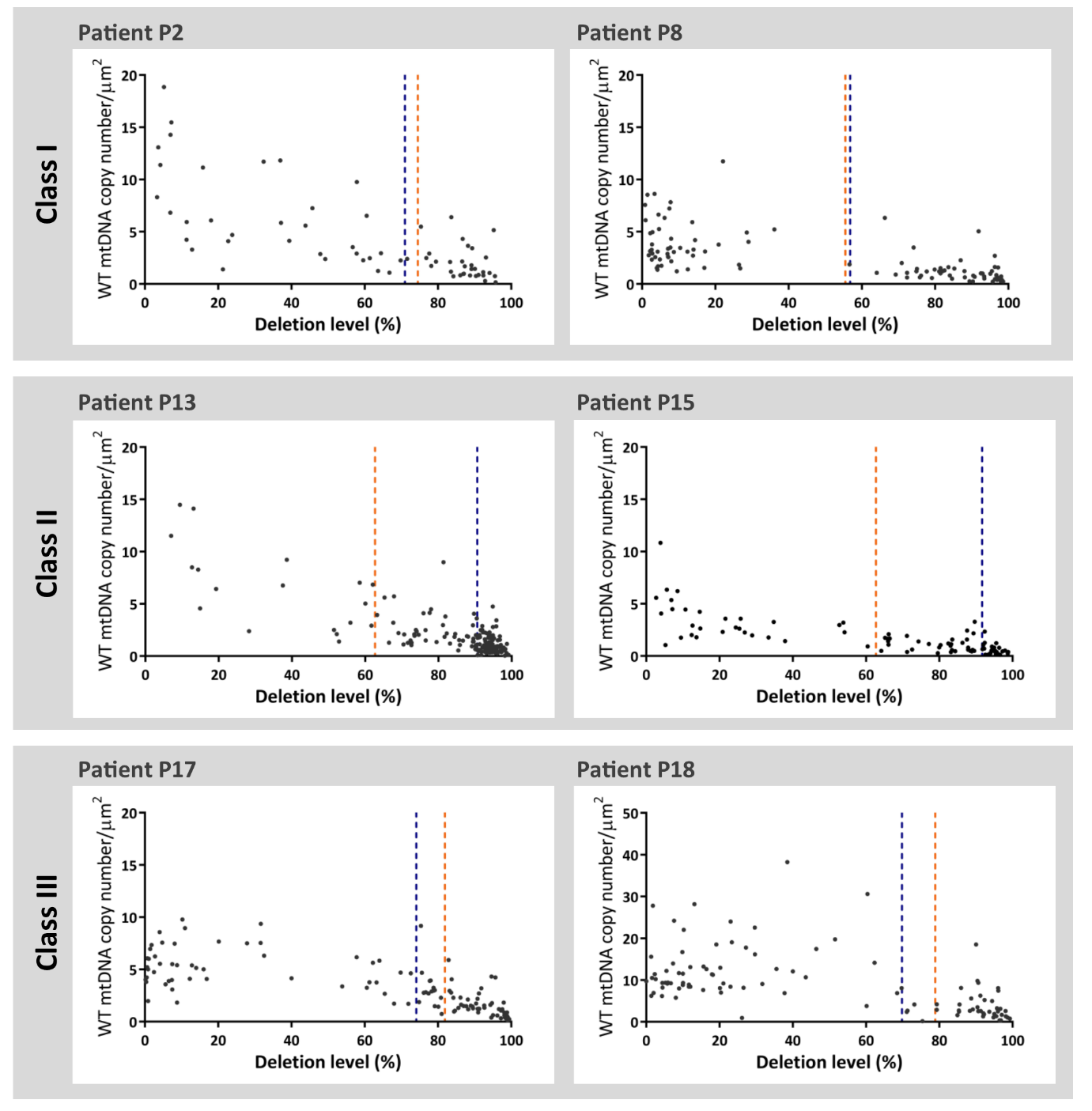


**Supplementary material for revision #2: Wild-type (WT) mtDNA copy number decreases after the biochemical threshold has been reached.** Deletion level and wild-type mtDNA copy number were plotted for each fibre. Each black dot represents one fibre. The previously determined biochemical threshold levels for complex I and IV are shown as orange and blue dashed lines, respectively.
